# Primary cardiac synovial sarcoma presenting with right heart failure and superior vena cava syndrome: a multimodal case report

**DOI:** 10.1097/RC9.0000000000000254

**Published:** 2026-03-02

**Authors:** Beyza Nur Su, Mehmet Furkan Sahin, Hayriye Tatli Dogan, Sena Gercek Civelek, H. Levent Mavioglu, Erdal Yekeler

**Affiliations:** a Department of General Thoracic Surgery and Lung Transplantation, Ankara City Hospital, University of Health Sciences, Ankara, Turkey; b Department of Pathology, Ankara City Hospital, University of Health Sciences, Ankara, Turkey; c Department of Cardiovascular Surgery, Ankara City Hospital, University of Health Sciences, Ankara, Turkey

**Keywords:** cardiac tumor, case report, intrapericardial, sarcoma, synovial sarcoma

## Abstract

**Introduction:**

Primary cardiac synovial sarcomas are extremely rare malignant tumors with nonspecific clinical features, often causing delayed diagnosis. Accurate differentiation from other cardiac neoplasms requires immunohistochemistry and confirmation of the SS18-SSX gene fusion via fluorescence in situ hybridization (FISH). Prognosis is poor, with 5-year survival around 35.7%. Multimodal treatment, including surgical resection and adjuvant therapy, is crucial to improve outcomes.

**Presentation of case:**

We report the case of a 45-year-old male who presented with a 2-month history of progressive dyspnea, orthopnea, and bilateral lower extremity edema. Imaging revealed a large anterior mediastinal mass compressing the heart, with posterior extension toward the pericardial surface and signs of superior vena cava syndrome. The patient underwent urgent surgical debulking via median sternotomy to relieve compression. No myocardial resection was performed; the tumor was dissected down to the epicardial surface. Histopathological examination confirmed a monophasic synovial sarcoma, and FISH analysis demonstrated the SS18-SSX gene fusion. Adjuvant chemotherapy was administered postoperatively. The patient experienced marked symptomatic improvement and remained clinically stable during follow-up.

**Discussion:**

Cardiac synovial sarcomas pose diagnostic and therapeutic challenges due to their rarity and aggressive nature. Surgical excision remains the cornerstone of treatment, although complete resection is often difficult. Molecular diagnostics play a critical role in confirming the diagnosis and guiding management. Adjuvant chemotherapy may contribute to symptom control and disease stabilization, though long-term survival remains limited. This case adds to the limited literature and underscores the importance of early recognition and multidisciplinary intervention.

**Conclusion:**

Primary cardiac synovial sarcoma should be considered in the differential diagnosis of cardiac masses presenting with unexplained dyspnea. Early diagnosis, surgical resection, and adjuvant therapy can lead to significant symptomatic relief and may improve survival. This case highlights the value of integrating molecular diagnostics into the routine evaluation of rare cardiac tumors.

## Introduction

Synovial sarcoma (SS) is a rare malignant soft tissue tumor arising from immature mesenchymal cells. Its gene expression profile is closely associated with neural crest-derived malignant peripheral nerve sheath tumors (MPNSTs)^[^1^]^. SS is most common between the ages of 15 and 40, with a male:female ratio of 1.2:1^[^1^]^. Histopathologically, SS can be monophasic, biphasic, or poorly differentiated^[^2^]^. Diagnosis has been facilitated by molecular techniques and cytogenetic methods^[^3^]^. A t(X;18)(p11.2;q11.2) translocation is found in over 95% of cases^[^1^]^. While over 70% occur in extremity soft tissues, rare sites include the kidney, bone, lung, and heart^[^4^]^. Intrapericardial SS is extremely rare; fewer than 20 primary cardiac cases have been reported in the last decade^[^5^]^.

HIGHLIGHTS

- Rare primary cardiac synovial sarcoma with intrapericardial localization.
- Multimodal imaging revealed severe cardiac compression, SUVmax 4.65.
- Diagnosis confirmed by IHC and FISH showing SS18 gene rearrangement.
- R1 resection plus adjuvant chemotherapy improved symptoms, no recurrence.
- Early surgery and multidisciplinary care are key for better PCSS outcomes.

This case report has been reported in line with the SCARE checklist^[^6^]^.

## Presentation of case

**Figure 1.**
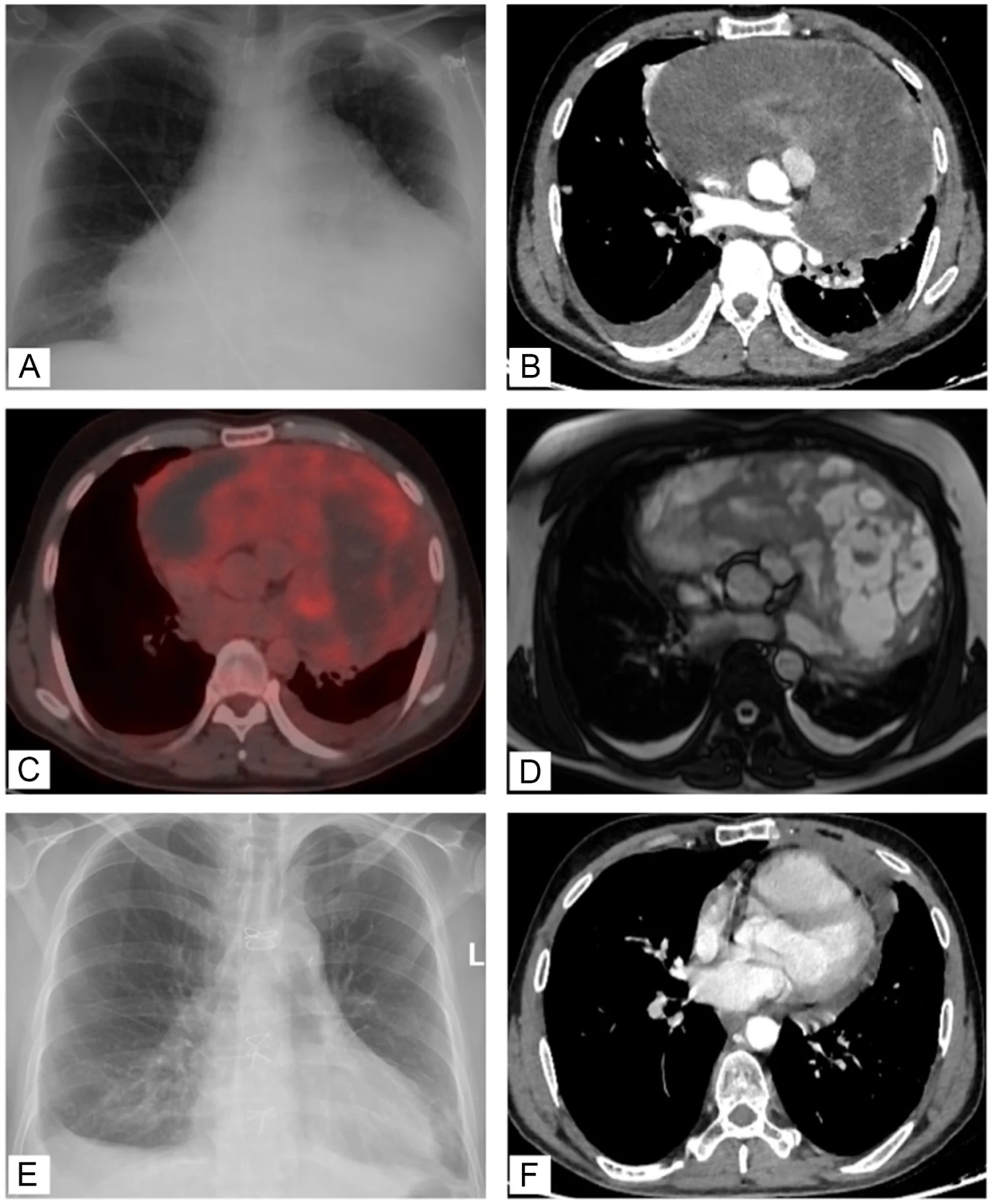
(A) Preoperative chest X-ray, (B) preoperative thorax CT, (C) preoperative PET/CT, (D) preoperative thorax MRI, (E) postoperative chest X-ray, (F) postoperative thorax CT. PET-CT, positron emission tomography-computed tomography; MRI, magnetic resonance imaging.

A 45–year-old male patient presented to our clinic with complaints of shortness of breath and swelling in his feet, which prevented him from lying on his back. He had no prior medical/surgical history, medications, allergies, family, or social risk factors. Clinically, the patient exhibited signs of superior vena cava syndrome and right heart failure, including orthopnea, bilateral pretibial edema, distended neck and chest wall veins, and progressive dyspnea. These findings were consistent with cardiac compression due to the mediastinal mass. An opacity filling the left diaphragmatic sinus was observed on the chest X-ray (Fig. 1a). Transthoracic echocardiography revealed a fibrinous pericardial effusion surrounding and compressing the heart, with an ejection fraction of 55%. Thoracic CT confirmed the presence of pericardial fluid and a large anterior mediastinal mass (Fig. 1b). Pericardiocentesis was performed for both diagnostic and symptomatic relief. Cytological analysis of the aspirated fluid was negative for malignancy and showed features consistent with acute inflammation. In cardiac magnetic resonance imaging (MRI), it was observed that ventricular filling was restricted due to mass compression, it created pressure, and the predominantly cystic heterogeneous cystic lesion was observed to be perfused in places with contrast material (Fig. 1d). In positron emission tomography-computed tomography (PET-CT), the standardized uptake value (SUVmax) of the lesion was calculated as 4.65 and no other tissue or organ involvement other than the mediastinum was observed (Fig. 1c). The result of mediastinotomy and biopsy, which were performed for diagnostic purposes before surgery, was interpreted as a malignant mesenchymal tumor, but detailed subtyping could not be made.

**Figure 2.**
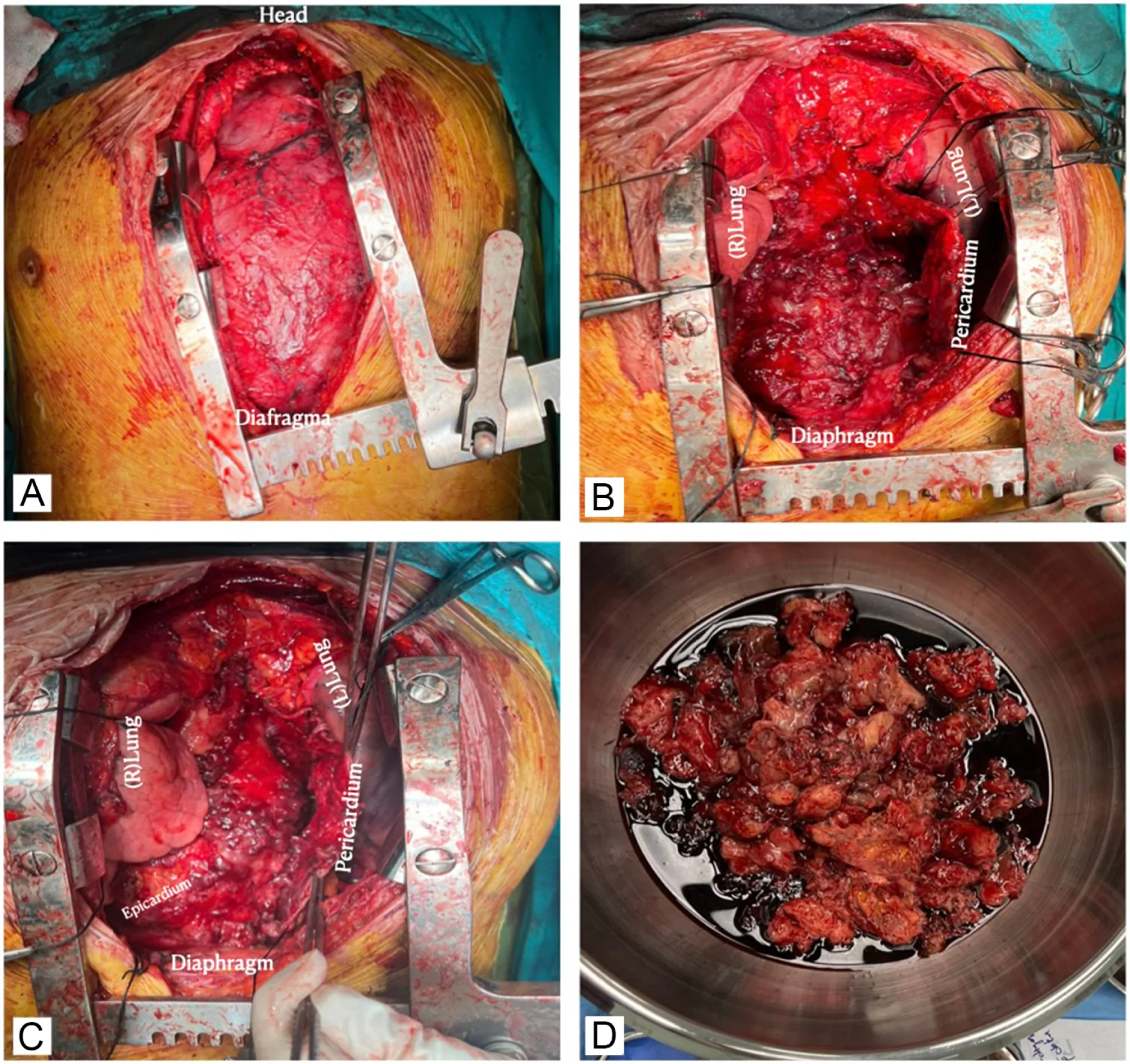
(A) Intraoperative view following median sternotomy, (B and C) operative field after tumor specimen, (D) macroscopic view of the tumor.

**Figure 3.**
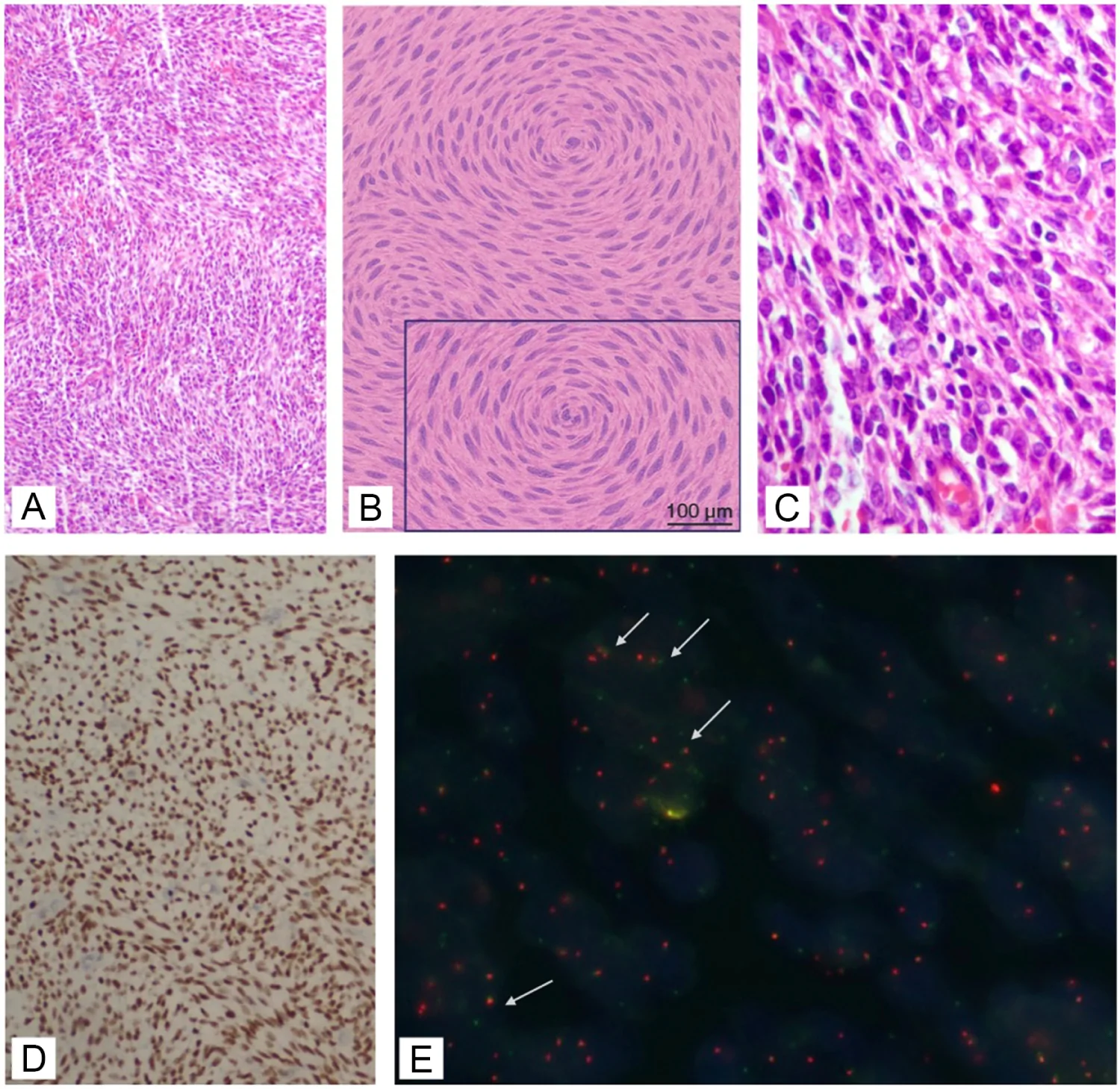
(A) Staining with hematoxylin-eosin (H&E) (×100), (B) prominent storiform and whorled patterns composed of spindle cells with mild nuclear atypia (H&E, ×200), (C) spindle-shaped tumor cells with focal nucleolar prominence (H&E, ×400), (D) immunohistochemical staining with TLE-1, (E) SS18 gene rearrangement by fluorescent in situ hybridization.

Given the rapid deterioration, the surgical approach was planned as urgent palliation. The goal was to relieve compression through debulking rather than achieve oncological clearance. The patient was approached through a median sternotomy (Fig. 2a). The mass was intraoperatively found to be a giant tumor originating from the myocardium at the level of the right ventricle, with an intrapericardial location and invading the ventricular wall. No myocardial resection was performed; the tumor tissue was dissected down to the epicardial interface. The procedure was classified as an R2 resection due to macroscopic residual disease (Fig. 2b-c). In the macroscopic examination, a fragmented mass was observed, which was fixed to the pericardium and removed together with the pericardium, measuring 30 × 25 × 5 cm in size, cream colored, and composed of fibrous tissues with areas of fat and necrosis tissue on its surface (Fig. 2d). In the histopathological examination, cellular mesenchymal neoplasia, characterized by spindle cells in a fascicular pattern, crossing each other, and sometimes in a hemangioperistematous pattern, was observed in the sections (Fig. 3c). In the immunohistochemical study, tumor cells stained positive for TLE-1 (Fig. 3d), CD56, CD99 (strongly membranous), EMA (focally weak) and SMA (focally weak); p63, S100, SOX10, PanCK, CK19, SALL-4,STAT-6, desmin, BCOR, SATB2, CyclinD1, ERG, chromogranin, synaptophysin, CD5, CD31, CD34, CD45, and TdT were stained negative. The mitosis index (Ki-67) was 25%. These neoplastic cells were detected as diffusely and strongly positive for TLE-1 (Fig. 3d) and CD99, and as focally positive for BCL2. Morphological and immunohistochemical findings were considered compatible with monophasic synovial sarcoma. The diagnosis was confirmed by the detection of SS-18 gene rearrangement in the fluorescence in situ hybridization (FISH) study (ss18 rearrangement rate: 70-80%) (Fig. 3e). In the early postoperative period, compression findings rapidly regressed, and dramatic improvement in the patient’s symptoms was observed and the patient was discharged on the 9th postoperative day (Table 1). The patient subsequently received six cycles of adjuvant chemotherapy consisting of a combination of ifosfamide, mesna and adriamycin, and no locoregional recurrence or distant metastasis was detected during the 61st postoperative week follow-up (Table 1) (Fig. 1e-f).

**Table 1:** Timeline of the case.

DATE	EVENTS
A few months before admission.	A 45-year-old male was suffered from dsypnea and edema.
	The previous physician detected cardiomegaly and requested an echocardiogram.
	Echo showed the massive pericardial effusion.
	Pericardiocentesis was performed.
	Symptoms do not improve and no definitive diagnosis could be established.
	The patient was referred to our clinic for further evaluation and treatment, as a mediastinal mass was suspected.
	Tumor resection was performed.
POD 1	Extubated.
POD 2	Taken to the inpatient ward.
POD 4	Echocardiography showed that the mass effect had resolved.
POD 9	He was discharge.
4 months follow-up	6 cycles of chemotherapy were completed.
A year follow-up	Clinically well with no recurrence and metastasis.

POD: Postoperative day

To clarify the timeline of the case, the patient presented with symptoms in March 2024, underwent surgery in June 2024, and completed six cycles of chemotherapy by September 2024. He remained recurrence-free and clinically stable during follow-up until August 2025 (Table 1).

## Discussion

Primary cardiac synovial sarcoma (PCSS) is an exceptionally rare entity, accounting for 4.2% of cardiac sarcomas and less than 1% of all primary cardiac tumors^[^7^]^. When the literature is reviewed, it is seen that 71% of the limited number of case reports include lesions arising from the right heart, mostly from the atrium^[^8^]^. In our case, the lesion appeared to invade all aspects of the heart, including the posterior aspect, rather than a single focus originating from the right ventricle. PCSS predominates in males and has a high incidence rate in males in their 30s. In the initial evaluation of PCSS, a heterogeneous mass covering the mediastinum accompanied by a pericardial and pleural effusion is usually observed on chest X-ray and CT and transthoracic echocardiography and cardiac MRI are helpful for detailed evaluation^[^4^]^. In a study evaluating PET-CT scans in cases of PCSS, it was emphasized that the patients’ SUVmax values ranged from 1.2 to 13.0 (median: 4.35) and disease-free survival was reduced. Additionally, it has been emphasized that the risks of local recurrence and metastatic disease are increased in patients with SUVmax values greater than 4.35^[^9^]^. In our case, imaging examinations showed that the SUVmax value of the heterogeneous lesion covering the mediastinum and compressing the heart was 4.65 on PET-CT.

The differential diagnosis of synovial sarcoma is broad due to its significant morphological and immunohistochemical heterogeneity. The biphasic variant, which contains epithelial components, may resemble sarcomatoid carcinoma, metastatic adenocarcinoma, biphasic mesothelioma, or MPNSTs with epithelial differentiation. Sarcomatoid carcinoma typically shows marked cytologic atypia, a pleomorphic cell population, and diffuse strong cytokeratin expression, whereas synovial sarcoma tends to exhibit focal or patchy cytokeratin/EMA positivity^[^2^]^.

The monophasic spindle cell variant, composed of uniform, narrow cytoplasmic spindle cells arranged in fascicles, may mimic fibrosarcoma, low-grade MPNST, leiomyosarcoma, myofibrosarcoma, or solitary fibrous tumor. Leiomyosarcomas typically show strong and diffuse SMA/desmin positivity, while MPNSTs exhibit focal and irregular S100 expression. These markers are usually negative or minimally expressed in synovial sarcoma. Additionally, the “staghorn” vascular pattern commonly seen in solitary fibrous tumors is not prominent in synovial sarcoma^[^2^]^.

Histologically, the characteristic whorled and tightly fascicular architecture, monomorphic appearance, and uniform oval nuclei of synovial sarcoma provide diagnostic clues. Immunohistochemically, TLE1 shows high sensitivity but limited specificity and should not be used in isolation. Definitive diagnosis relies on molecular confirmation of the t(X;18)(p11;q11) translocation, indicating SS18-SSX fusion, which remains the gold standard for distinguishing synovial sarcoma from morphologically similar tumors^[^4^]^. In our case, the tumor exhibited diffuse TLE1 and CD99 positivity (Fig. 3d), with negative staining for S100, CD34, and calretinin. FISH analysis confirmed the presence of SS18-SSX fusion, supporting the diagnosis of monophasic synovial sarcoma (Fig. 3e).

PCSS is an aggressively progressing tumor and has a poor prognosis. Considering its rarity, no consensus has yet been reached on the treatment of PCSS. For these aggressive lesions, which are known to have a high probability of recurrence, a wide surgical resection that provides negative tumor margins forms the main basis of the treatment strategy^[^8^]^. Postoperative chemotherapy, radiotherapy, or concurrent chemoradiotherapy is usually the preferred multimodal treatment approach^[^10^]^. Less than a quarter of the published cases have been found to be completely resectable due to anatomical location and aggressive spread. However, systemic chemotherapy after surgery is thought to be beneficial for disease-free survival and overall survival in advanced unresectable cases or in patients in whom R0 resection could not be achieved.^[^11^]^. There are only 54 published articles describing cardiac synovial sarcomas, with a total of 60 patients. The median survival is approximately 24 months, and 1-year and 5-year survivals are 59.9% and 29.9%, respectively, with chemotherapy and age significantly affecting overall survival^[^12^]^.

When reviewing the literature, a few reports have documented primary cardiac synovial sarcoma requiring emergency surgery. One reported case involved an elderly Chinese woman with a primary synovial sarcoma originating in the right heart and affecting the tricuspid valve. She underwent surgical excision of the mass and survived the event^[^13^]^. Another published case described a young Egyptian male in whom the tumor filled the right atrium and intermittently extended into the right ventricle. He likewise received urgent surgical removal of the lesion with initial success, but later died as a result of intracranial hemorrhage secondary to brain metastasis^[^14^]^ Notably, the longest postoperative survival documented in the literature is 14 years, achieved in a patient who received multimodal treatment consisting of surgery, chemotherapy, and radiotherapy^[^15^]^.

In our case, due to the tumor’s multifocal infiltration and epicardial adherence, complete resection was not feasible. No myocardial tissue was excised; instead, surface-level debulking was performed to relieve cardiac compression. Therefore, the procedure was classified as an R2 resection. The patient’s symptoms improved markedly in the early postoperative period, with normalization of heart rhythm and resolution of lower extremity edema. He received six cycles of adjuvant chemotherapy, and no recurrence or metastasis was observed during 61 weeks of follow-up (Table 1).

This case aligns with previously reported PCSS cases, of which fewer than 20 have been documented in the past decade. Most arise from the right heart, and complete resection is rarely achievable. Prognostic factors associated with poor outcomes include tumor size >5 cm, male gender, extensive necrosis, incomplete resection, SYT-SSX1 fusion, and high mitotic index (>10/HPF)^[^4,12^]^. Our patient had several of these risk factors, yet remained stable postoperatively, likely due to timely palliation and multidisciplinary care.

## Conclusion

Surgical debulking can be a valuable component of multimodal therapy in patients with cardiac compression due to PCSS, even when complete resection is not feasible. In such cases, palliative surgery may provide significant symptomatic relief and facilitate timely oncological referral. This case underscores the importance of early recognition, molecular diagnostics, and multidisciplinary management in rare cardiac tumors.

The patient expressed gratitude for the multidisciplinary care and reported significant improvement in his quality of life following surgery and chemotherapy. He remains under regular follow-up and has resumed daily activities.

## Data Availability

The datasets generated during the current study are available from the corresponding author upon reasonable request.
